# An analysis of non-cultivable bacteria using WEKA

**DOI:** 10.6026/97320630016620

**Published:** 2020-08-31

**Authors:** Pritee Chunarkar Patil, Pradnya Suresh Panchal, Shweta Madiwale, Vidya Sunil Tale

**Affiliations:** 1 Department of Bioinformatics, Rajiv Gandhi Institute of IT and Biotechnology, Bharati Vidyapeeth Deemed University, Pune, Maharashtra, India

**Keywords:** Metagenomics, T-RFLP, Uncultured microorganisms, WEKA, SMO algorithm

## Abstract

The study of metagenomics from high throughput sequencing data processed through Waikato Environment for Knowledge Analysis (WEKA) is gaining momentum in recent years. Therefore, we report an analysis of metagenome data generated using T-RFLP followed by using the SMO (Sequential minimal optimization) algorithm in WEKA to identify the total amount of cultured and uncultured microorganism present in the sample collected from multiple sources.

## Background

Multiple methods have been developed to culture different kinds of microorganisms. However, the availability of complex growth medium has helped in isolating only 1% of microorganisms from distinct habitats. Thus, 99% of microorganisms are still not cultured [1]. Metagenomics helps to uncover these unknown species [2] Sequencing is important for metagenomics analysis especially where species are not indetified. Sequencing technologies has revolutionized Biology [3]. Low cost of sequencing, advances in the field of Bioinformatics, development of data library, tools and databases for metagenome has impact on metagenomics study [1]. A wealth of information has been obtained using metagenomics such as microbial diversity, uncharacterized metabolisms and increased complexity of biogeochemical pathways [2].

## Cultured and uncultured organisms:

The number of cells that were observed microscopically far outweighed the number of colonies that grow on a petri plate [4]. The uncultured microorganisms are the rich source of secondary metabolites; they can also be involved in commercial production of enzyme. The uncultured microorganisms also have many industry applications, which includes identification of novel biocatalysts, development of new antibodies, bioremediation etc. [2,5]. Hence it is important to study uncultured microorganisms to understand their biology in detail. Therefore, it is of interest to isolate the DNA from water samples. Samples were collected from multiple fresh water reservoirs in Pune, India. The amount of the DNA extracted was further analyzed by T-RFLP method, which falls between conventional DNA sequencing and next generation sequencing. It amplify 16S rRNA by digesting it through specific restriction enzymes. After digestion, it generates number of peaks, which is equivalent to number of strains present in given sample with the help of capillary electrophoresis. T-RFLP will generate ample amount of data for further processing through bioinformatics techniques. It is of interest to identify the uncultivable bacterial species.

## WEKA Analysis:

The Waikato Environment for Knowledge Analysis commonly known as WEKA provides a comprehensive collection of Machine Learning algorithms and data preprocessing tools for research community. It is a freely available software package funded by New Zealand government in 1993. It allows user to compare different machine learning algorithms on data set. Data mining is the extraction of hidden predictive information from large databases and it is a powerful new technology with great potential to help user focus on the most important information in the data warehouses. Data mining tools predict future trends and behaviors, allowing businesses to make proactive and knowledge-driven decisions. Many new data mining algorithms have been added since WEKA has been developed [6,7].

## WEKA utility:

[1] Free availability under the GNU General Public License.

[2] Portability. It is fully implemented in the Java programming language and thus runs on almost any modern computing platform

[3] A comprehensive collection of data preprocessing and modeling techniques

[4] Ease of use due to its graphical user interface

WEKA supports several standard data mining tasks, more specifically, data preprocessing, clustering, classification, regression, visualization, and feature selection [6, 7].

## SMO Algorithm:

Support Vector Machine (SVM) is the most commonly used machine-learning algorithm for classification because of its high precision. But there are very few researchers who actually work on SVM. These is mainly because of the complexity of the algorithm or the slow training set algorithm of SVM. To overcome this drawback, a new algorithm has been developed which is known as Sequential Minimal Optimization (SMO) algorithm. Fast SVM training speed with SMO algorithm is an important goal for practitioners. SVM learning algorithm has used Numerical Quadratic Programming while SMP has used an analytical Quadratic Programming [8,9].

## Methodology

### Sample Collection:

Fresh water samples were collected from multiple fresh water reservoirs from Pune city, India. A list of all water reservoirs is mentioned in Table 1.

### DNA Isolation:

The next step after sample collection is isolation of genomic DNA from sample. Physical separation and isolation of cells from the samples might also be important to maximize DNA yield or avoid co-extraction of enzymatic inhibitors that might interfere with subsequent processing. Certain types of samples (such as biopsies or groundwater) often yield only very small amounts of DNA. Library production for most sequencing technologies require high nanograms or micrograms amounts of DNA and hence amplification of starting material might be required [10]. After isolation of genomic DNA, Quantitative and Qualitative analysis was carried out with the help of agarose gel electrophoresis and spectrophotometer respectively.

### PCR Amplification and Purification:

Now before sequencing the DNA, it was important to amplify the DNA sample since the amount of DNA yield after isolation was very low. Amplification was carried out using PCR technique. PCR products were purified using Purelink PCR purification kit (Invitrogen, USA). PCR product purification is essential to be as it removes unused PCR components, dNTPs and excess primers from the amplicons. The purification is essential to be done since the impurities might hinder the sequencing reaction. After purification the samples are again checked on 1% agarose gel to see whether the eluted product contains the necessary amplicons in sufficient concentration. After this the products are taken for DNA sequencing using the BigDye Terminator V 3.1 Cycle sequencing kit (Applied Biosystems, USA).

### DNA Sequencing:

DNA Sequencing is carried out using BigDye Terminator Cycle Sequencing Kit developed by applied Biosystems. The BigDye® Terminator v3.1 Cycle Sequencing Kit's robust, highly flexible chemistry is ideal for de novo sequencing, resequencing, and finishing with PCR product, Plasmid, Fosmid, and BAC templates. The main aim of the project is to identify total amount of culturable and unculturable microorganisms using bioinformatics tools-BLAST and WEKA. The sequencing of genomic DNA has generated total 3370 sequencing reads, out of which 1001 sequences were selected for further analysis using BLAST and WEKA.

### BLAST search:

Basic Local Alignment Search Tool (BLAST) most commonly used sequence analysis tool available on Nation Center for Biotechnology Information. BLAST is sequence similarity search program. BLAT was used to identify culturable microorganisms from the sample using sequence similarity method [11]. The BLAST output was then further verified using WEKA software.

### Input data for WEKA:

**Figure 1:**
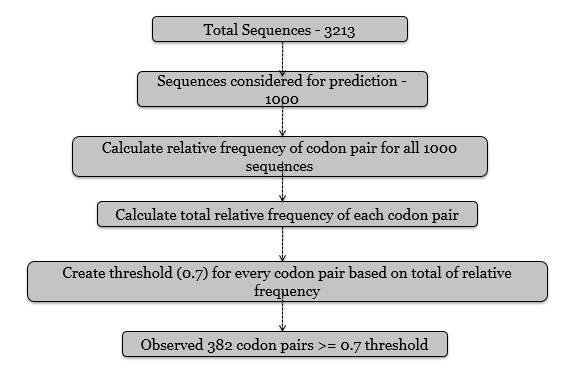
Flowchart of the steps used in WEKA input file generation

As shown in Figure 1, for WEKA analysis, Codon-pair feature is used as a factor for prediction and classification of sequences. Relative frequency of each codon-pair for all the sequences is calculated. For prediction through codon-pair factor as the number of codon-pairs increases the prediction or classification precision decreases. Hence, a threshold of 0.7 is set that is only the codon-pairs having total relative frequency above 0.7 are further considered as classification features. According to the set threshold out of 4096 codon-pairs, 382 codon-pairs are considered for precise classification of sequences. Then based on this observation, Attribute Relation File Format (ARFF) file is prepared as an input file for WEKA analysis.

### SMO algorithm:

**Figure 2:**
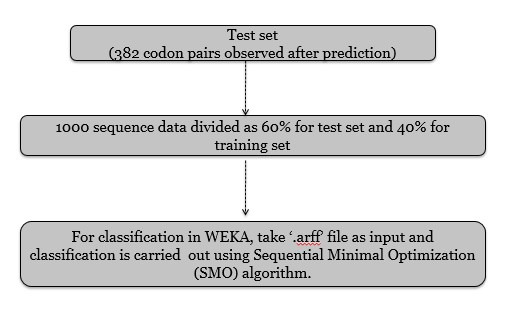
Flowchart of steps used in classification of data

In WEKA the algorithm used for classification is Sequential Minimal Optimization (SMO) algorithm. As shown in Figure 2, the data is now classified as test set and training set for applying the algorithm. SMO Algorithm is used to predict the correctness of classification of culture and uncultured microorganisms.

## Results and Discussion:

**Figure 3:**
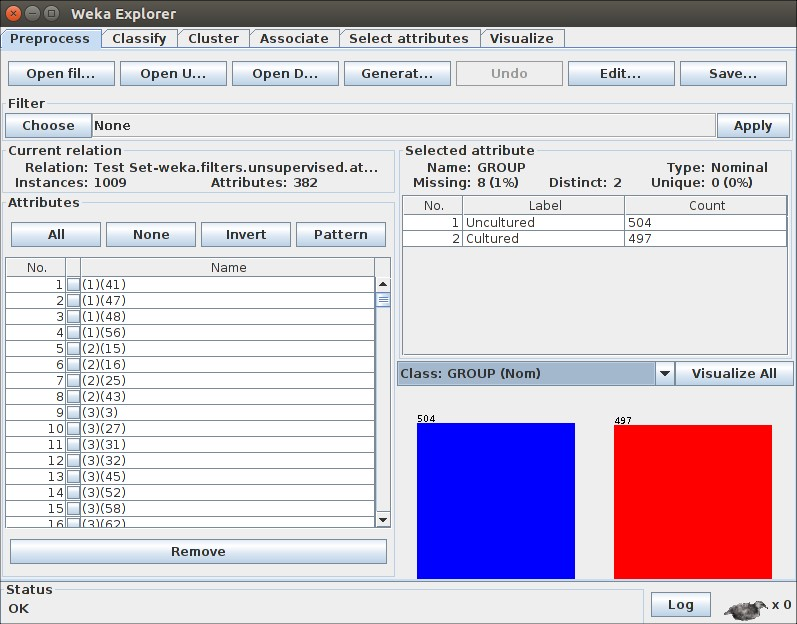
Input file in WEKA

**Figure 4:**
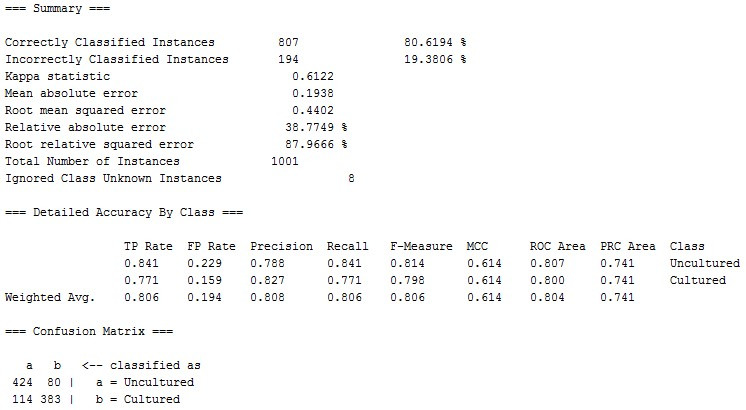
Output summary of SMO analysis

A dataset having 1001 genomic DNA sequences were filtered from a dataset of 3779 genomic DNA by applying a threshold of 0.7 relative frequencies for every codon pair in the dataset. This dataset was further analyzed for classification using Sequential Minimal Optimization (SMO) in WEKA. The dataset was divided into two groups namely cultured and uncultured microorganisms. As result shown in Figure 3, 807 instances was shown to be correctly classified with a significant 80.62% accuracy using SMO algorithm. The SMO constructed the below result for given input file. Figure 4 shows that 80.6194% of instances were correctly classified. It simply means that out of 1001 sequences 807 sequences were correctly classified.

## Conclusion

We report the grouping of cultured and uncultured microorganism in metagenomics by analyzing their T-RFLP data using the SMO algorithm in WEKA. It indicated the groups very clearly. The same program could be utilized for the identification of physical metagenomics sequences via kit with the help of biomarkers identified from uncultivable microorganisms.

## Declaration on Publication Ethics:

The authors state that they adhere with COPE guidelines on publishing ethics as described elsewhere at https://publicationethics.org/. The authors also undertake that they are not associated with any other third party (governmental or non-governmental agencies) linking with any form of unethical issues connecting to this publication. The authors also declare that they are not withholding any information that is misleading to the publisher in regard to this article.

The authors are responsible for the content of this article. The Editorial and the publisher has taken reasonable steps to check the content of the article with reference to publishing ethics with adequate peer reviews deposited at PUBLONS.

## Figures and Tables

**Table 1:** List of water Reservoirs

Rivers (108 Samples from 12 different Locations)	Lakes (36 Samples, 9 each)	Dams (65 Samples)
Mula	Venna Lake	Khadakwasla Dam
Mutha	Pashan Lake	Panshet Dam
	Katraj Lake	Warasgaon Dam
	Vishrantwadi Lake	Temghar Dam
		Mulshi Dam
		Bhatghar Dam
		Varasgaon Dam
